# Histological and Clinical Investigations as to the Method in Which Nitrate of Silver Affects Carious Dentine

**Published:** 1902-05

**Authors:** William H. Potter


### Reviews of Dental Literature.

Histological and Clinical Investigations as to the Method in which Nitrate of Silver affects Carious Dentine. By Dr. Josef Szabó, Budapest.[1]

The author gives a very carefully prepared history of the uses of nitrate of silver in dental practice beginning with the year 1846, and quoting leading exponents of the drug. He then comes to the question, What is its penetration power, and what is its chemical action on the fibrillæ? As to this power, there has been considerable divergence of opinion, some holding that it could not penetrate into the tubules, and others holding that this power was so great that the pulp might be endangered. Investigations as to the penetrating power of nitrate of silver have heretofore been made upon extracted teeth. The author, however, considers that extracted teeth may react differently with the drug than teeth which are in the mouth. He, therefore, makes his experiments upon living teeth. A right and a left lower first molar having a crown cavity is selected. The rubber dam is adjusted, and the cavities are dried out and excavated as far as possible. The nitrate of silver is then applied in different forms,—viz., in powder, as a ten, twenty, thirty, and forty per cent. solution. The applications were made five, fifteen, eighteen, and twenty-five times. After the experiment was performed the tooth was extracted and examined under the microscope. Pictures are given of some of these examinations, and show clearly the penetration of the drug when in contact with living dentine. "Without regard to the concentration or method of use, we find the same result. The carious dentinal tubules are filled up to a certain depth, with a coarsely lumped contents." According to these observations the tubules are affected only to a certain depth; when this is reached further applications of the drug cannot force it deeper.

[1] Histologische und klinische Untersuchungen über die Wirkungsweise des Argentum nitricum auf das ehrankte Dentin. Von Dr. Josef Szabó, Osterreichisch-ungarische Vierteljahrsschrift für Zahnheilkunde, Jänner, 1902.

The infiltrated dentine layer was about one-half millimetre thick. A deeper penetration could never be observed, even though the most diverse solutions be used.

In regard to the question as to how nitrate of silver acts upon the contents of the dentinal tubules, there is this explanation given: "The living albumin is changed, under the action of the silver salt, to dead albumin. The plasma is changed from a soluble condition to a solid mass. The albumin unites itself in the form of a precipitate with the metal of the silver salt. The finely granular albuminate of silver is easily recognizable under the microscope, and shows its characteristic properties. Under the action of light it becomes dark, then black and insoluble."

The author, in concluding, says that he must agree with Walkhoff that nitrate of silver has a relatively slight power of penetration and does not act deeply enough to endanger the vitality of the fibrillæ.

William H. Potter.

